# Powdered human milk‐derived versus bovine milk‐derived breastmilk fortification: A multi‐centre preterm randomised controlled trial

**DOI:** 10.1002/jpn3.12431

**Published:** 2024-12-11

**Authors:** Janet Berrington, Mark Johnson, Shalabh Garg, Christopher Stewart, Christopher Lamb, Jeremy Palmer, Nicholas Embleton

**Affiliations:** ^1^ Newcastle Neonatal Services Newcastle upon Tyne Hospitals NHS Foundation Trust Newcastle upon Tyne UK; ^2^ Translational and Clinical Research Institute Newcastle University Newcastle upon Tyne UK; ^3^ Department of Neonatal Medicine Princess Anne Hospital Southampton UK; ^4^ NIHR Southampton Biomedical Research Centre University Hospital Southampton NHS Foundation Trust and University of Southampton Southampton UK; ^5^ Neonatal Unit James Cook University Hospital Middlesbrough UK; ^6^ Population Health Sciences Institute Newcastle University Newcastle upon Tyne UK

**Keywords:** calprotectin, growth, inflammation

## Abstract

**Objective:**

To compare faecal calprotectin, plasma amino acids and clinical outcomes in preterm infants receiving powdered human milk‐based fortifier (PHMF) compared to powdered bovine milk‐based fortifier (PBMF) in preterm infants on an otherwise exclusive human milk diet.

**Methods:**

A randomised controlled trial in infants <32 weeks of gestation or <1500 g who only received human milk and had reached full enteral feeds (150 mL/kg/day), without pre‐existing gastrointestinal morbidity. Primary outcome was faecal calprotectin within 21 days of starting fortification; secondary outcomes were calprotectin at discharge, plasma amino acids and clinical outcomes, including growth and neonatal morbidities.

**Results:**

The trial stopped early after the manufacturer's withdrawal of PHMF. Thirty‐one infants were enroled, three without informative sampling, leaving 14 per group. No statistical differences were seen in faecal calprotectin on Day 7 (236 mcg/g PHMF vs. 303 mcg/g PBMF, *p* = 0.90) or 21 (135 mcg/g PHMF vs. 315 mcg/g PBMF, *p* = 0.21). Adjusting for gestation and day of life, and including all time points after enrolment to discharge, fortifier type did not impact faecal calprotectin (coefficient estimate −7.13, 95% confidence interval = −172 to 158, *p* = 0.93). Rates of key neonatal morbidities did not differ. PHMF infants grew more slowly reaching statistical significance in change in weight standard deviation score at discharge compared to PBMF infants (mean (standard deviation) −0.94 (0.7) PHMF vs. −0.24 (0.8) PBMF, *p* = 0.02).

**Conclusions:**

We did not detect reduced gut inflammation as measured by faecal calprotectin in PHMF compared to PBMF but weight gain was slower, of potential clinical importance.

## Abstract (highlights)

**What is Known:**

- Human milk is associated with improved survival and lower rates of necrotising enterocolitis, but growth without fortification is challenging.
- Fortification previously relied on powdered bovine milk‐derived products (thus introducing bovine proteins) or liquid human milk‐derived products (thus displacing some maternal milk). Powdered human milk‐derived fortifier avoids these two issues.

**What is New:**

- This is the first report of a randomised controlled trial of powdered human milk‐derived fortification (PHMF) compared to powdered bovine milk‐derived fortification (PBMF) in infants <32 weeks of gestation.
- PHMF did not reduce gut inflammation (measured by stool calprotectin), but the weight standard deviation score at discharge was significantly further from baseline compared to infants fortified with PBMF.

## Graphical abstract

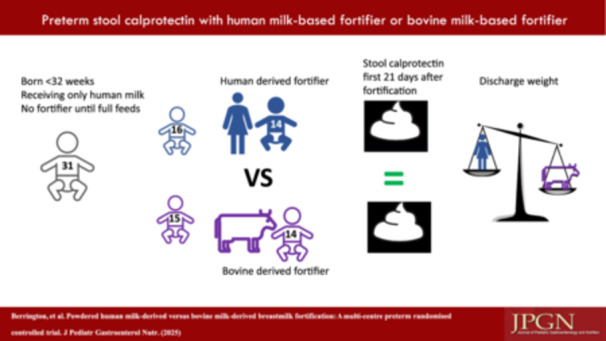

## 1 INTRODUCTION

Preterm birth below 32 weeks of gestation is associated with increased risks of death, physical and cognitive impairment^1^ and increased healthcare costs.^2^ Survival and morbidities such as necrotising enterocolitis (NEC) are influenced by diet, with morbidity and mortality lower in infants with higher intakes of mother's own breastmilk (MOM).^3^ When there is insufficient MOM, the shortfall can be provided using bovine milk‐based formula or pasteurised, donated human milk (DHM).^4^, ^5^

Human breast milk alone does not meet the nutrient requirements of these infants and fortifiers are widely used^6^, ^7^, ^8^ since poor growth is linked to worse outcomes.^9^ Most fortifiers are powdered and from bovine sources (powdered bovine milk‐based fortifier, PBMF). Concern exists that bovine products are harmful but strong data on the risks from bovine fortifiers in an otherwise exclusive human milk diet (EHMD) do not exist.

Liquid human milk‐based fortifiers (LHMF) have been used in the United States for >10 years.^10^ They avoid bovine proteins and provide important human bioactive constituents,^11^ but processing loses some constituents.^12^ The commonest LHMF (humavent +6 Prolacta™) is recommended in a 35 mL MOM to 15 mL fortifier ratio displacing 30% of MOM, potentially reducing benefits. Before our study commenced, one randomised controlled trial (RCT) had compared PBMF with LHMF in preterm infants receiving EHMD, finding no differences in faecal calprotectin, clinical outcomes or growth among 127 infants.^13^ Recently, powdered HMF (PHMF) has been developed that avoids displacing any MOM.

Preterm infants receiving only unfortified MOM compared to any other diet have been shown to have lower calprotectin levels.^14^ To explore the impact on gut inflammation of PHMF compared to PBMF in infants receiving EHMD we chose faecal calprotectin as the primary outcome, hypothesising lower faecal calprotectin in PHMF infants. Calprotectin is a member of the S100 family of proteins found in neutrophils, monocytes and macrophages, and in stool when released by granulocytes in the presence of gut inflammation. Secondary outcomes reported here include key neonatal morbidities, including growth and plasma amino acids, given the different protein sources between fortifier types. Additional secondary outcomes (stool microbiome cytokines and immunoglobulin A [IgA] and urine IgA) will be reported separately.

## 2 METHODS

In a randomised, open‐label, controlled trial, we enroled infants <32 weeks of gestation or <1500 g across two sites who had received EHMD (MOM or DHM or both) and not commenced fortification. We followed the Consolidated Standards of Reporting Trials (CONSORT) reporting guideline (Figure S1). Infants were randomised when tolerating full feeds (150 mL/kg/day) for ≥24 h, and excluded if they had: already received formula or fortifiers, congenital abnormalities affecting feeding, bowel surgery or NEC before randomisation, or the clinician did not wish to fortify. Fortification began after full enteral feeds to separate intolerance from fortifier from intolerance due to volume increases. The study was approved by the Sheffield Research Ethics Committee (ref 21/YH/0224) and registered prospectively (ISRCTN 22484792). Three neonatal units participated, and two recruited infants; one did not have internal permissions in place before product withdrawal occurred. The full protocol is available (Supporting Information S1: Protocol).

We hypothesised that PHMF (intervention) compared to PBMF (control) would reduce faecal calprotectin. The primary outcome was faecal calprotectin analysed at Days 7 and 21 after the start of fortification. Based on faecal calprotectin values in 142 healthy infants of 4.34 ± 1.94 mg/dL, and to determine a 50% reduction in faecal calprotectin levels with 80% power and 5% significance, 36 infants (18 per group) were needed.^15^ Allowing for losses and incomplete sampling we aimed to recruit up to 50 infants until 36 cases with sampling over the first 21 days (informative) were obtained.

Parents were approached by the study team, given a parent information sheet and provided written informed consent (Supporting Information S1: Protocol). We established a Trial Steering Committee (TSC) that included parent representation. Two amendments were approved: (1) an extension to the end date of 6 months to 30/06/2023 and (2) suspension of the study after PHMF product withdrawal (December 2022).

Randomisation used a secure web‐based service (Sealed Envelope Ltd) with site and gestation <28 weeks strata. Twins were allocated the same intervention, only randomising the first. Infants were fortified as per standard unit practice in both groups (introduced as 50% of milk volume fortified moving to 100% of milk volume fortified once tolerated) and fortification was not individualised or blinded: the intervention group using commercially available powdered PHMF (Neokare Ltd) and control group using commercially available PBMF (Nutriprem [Nutricia Ltd] or SMA Fortifier [SMA nutrition] determined by unit choice). Vitamin and mineral supplements were recommended in the trial protocol to ensure equality across groups guided by routine laboratory results. All milk given for at least the first 21 study days was human, either MOM (preferentially) or DHM, used only in the event of MOM shortfall. After the first 21 study days, supplemental milk type was at the principal investigator's discretion. Infants remained on assigned fortifier until discharge, transfer or 36 weeks corrected gestation, study withdrawal or suspension, whichever was sooner.

We collected demographic and outcome data, including gestation, birthweight, mode of delivery, antenatal steroid receipt, antenatal Doppler concerns, weights and head circumferences at enrolment, Days 7, 14 and 21 after fortification and at discharge, number of days fortified, days where feeds were paused for more than 4 h (and reason), antibiotic exposure (days), days when stool was passed, NEC (medical or surgical), focal intestinal perforation (FIP), milk curd obstruction (MCO, surgically diagnosed), late‐onset sepsis (LOS [blood culture positive; identified organism]), retinopathy of prematurity (ROP) requiring treatment, and intraventricular haemorrhages (IVHs) or cystic periventricular leukomalacia (PVL) identified on ultrasound. Secondary clinical outcomes were recorded on standardised forms defining all outcomes, including grade of NEC from IA to IIIB, details of timing and organisms for LOS, and grades of IVH and presence of PVL and post‐haemorrhagic hydrocephalus (Supporting Information S1: Appendix 6).

We aimed to collect two stools (to prevent a freeze/thaw cycle for secondary analyses) at enrolment, Days 7, 14 and 21 days after fortification started and at 36 weeks post‐menstrual age (PMA) or transfer/discharge if sooner. Samples were frozen locally and transferred on dry ice to Newcastle NHS laboratory for analysis of calprotectin by ELISA (enzyme‐linked immunosorbent assay) in one single run. Targeted blood was taken for amino acids (secondary outcome) on Days 7 and 21, analysed locally using ion exchange chromatography by the Biochrom 30+ at both sites (Southampton and Newcastle).

### 2.1 Statistical analysis

The chi‐square test and Mann–Whitney *U* test were used to compare proportional and quantitative values, respectively. Spearman's correlation was used to evaluate the relationship between infant characteristics (day of life, day after fortification started, gestation, total days fortified and PMA) and faecal calprotectin; variables with a statistically significant correlation with faecal calprotectin by univariate analysis were subsequently analysed in multivariate regression alongside trial arm (GraphPad Prism 9.41). Statistically significant differences are assumed when *p* < 0.05.

## 3 RESULTS

From 50 approached infants, we enroled 31 between April 2022 and December 2022, when the study was suspended due to manufacturer withdrawal of the PHMF product. Sixteen were randomised to PHMF and 15 to PBMF. One infant did not provide samples over the first 21 days (PBMF), one was recruited on the day the study stopped (PHMF) and one was withdrawn by the clinician after loss of equipoise (PHMF) leaving 14 infants in each group for analysis (Figure S1 Consort Diagram). Demographic data were well balanced between arms (Table 1). Median days fortified, tolerance and days nil by mouth did not differ statistically between groups. PHMF infants grew more slowly, with the change in weight standard deviation score (SDS) from enrolment to discharge being significantly different between groups (*p* = 0.02). No infant experienced intestinal perforation or gastrointestinal surgery. One episode of medical NEC occurred on study Day 8 in the PHMF group with full recovery. Other pre‐specified clinical outcomes did not differ statistically between groups (Table 2).

**Table 1:** Baseline characteristics of study groups.

	Control PBMF (*n* = 15)	Intervention PHMF (*n* = 16)
Gestation (weeks)^a^	27.3 (26.1–30.4)	27.6 (24.3–28.8)
Birthweight (g)^a^	1040 (810–1480)	955 (633–1288)
Birthweight standard deviation score^a^	0.13 (−0.66 to 0.87)	0.41 (−0.97 to 0.93)
Multiple pregnancy^b^	0	2 (12.5)
Male sex^b^	7 (47)	9 (57)
Caesarean delivery^b^	11 (73)	9 (57)
Antenatal steroids (any)^b^ [complete course]^b^	15 (100) [10 [67]]	15 (94) [9 [56]]
Antenatal absent/reversed end‐diastolic flow^b^	2 (13)	2 (12.5)
Rupture of membranes >24 h^b^	3 (20)	5 (31)
Ethnicity—non‐white British^b^	3 (20)	1 (6)
Age at enrolment (days)^a^	12 (10–17)	15.5 (12–22)
Corrected gestation at enrolment^a^	30 (28.6–32)	29.9 (26.6–30.8)

*Note*: Data are median (IQR) (a) or *n* (%) (b). Includes three infants randomised but without informative samples. One infant in each group died from CLD, not considered due to study intervention. There were no serious adverse events not pre‐specified in the protocol, but termination due to product recall of the PHMF was not foreseen.

Abbreviations: CLD, chronic lung disease; IQR, interquartile range; PBMF, powdered bovine milk‐derived fortification; PHMF, powdered human milk‐derived fortification.

**Table 2:** Clinical outcomes by study group.

	Control PBMF (*n* = 14)	Intervention PHMF (*n* = 14)	*p*
First 21 days of study			
Days of fortifier use^a^	21 (14–21)	21 (21–21)	0.07
Infants receiving donor milk	3 (21)	6 (42)	0.42
Days donor milk use^a^	0 (0–0)	0 (0–19)	0.17
Days nil by mouth for >4 h^a^	0 (0–0)	0 (0–0)	>0.99
Days when stool passed^a^	19 (13–21)	21 (20–21)	0.06
Days of antibiotics^a^	0 (0–3)	3.5 (0–7.5)	0.08
Blood culture positive LOS^b^	0	2 (14)^f^	0.22
Change in SDS enrolment to Day 21			
Weight^d^	−0.34 (0.47)	−0.72 (0.5)	0.08
Head circumference^c^	−0.29 (0.79)	−0.48 (1.0)	0.68
Predischarge/transfer data			
NEC^b^ [surgical NEC]^b^	0	1 [0]	0.43
Blood culture positive LOS^b^			
Day 22 to discharge from study	1 (6.6)^e^	2 (14)^g^	0.60
ROP requiring treatment^b^	2 (13)	1 (7)	>0.99
Intraventricular haemorrhage (any)^b^	2 (13)	3 (21)	0.65
Died before discharge home^b^	1 (6.6)	1 (7)	>0.99
Days in study before 36 weeks PMA, discharge or death^a^	34 (14–41)	38 (28–48)	0.13
PMA at discharge home (weeks)^a^	37.4 (35.8–39.1)	38.9 (37.4–40.8)	0.26
Milk at discharge or death^b^			
Any maternal	11 (79)	12 (86)	0.99
Exclusive maternal	11 (79)	7 (50)	0.26
Any formula	3 (21)	1 (7)	0.59
Any donor	0	5 (35)	0.04
Change in SDS enrolment to discharge^d^			
*Weight^c^	−0.24 (0.8)	−0.94 (0.7)	0.02
Head circumference^c^	−0.03 (1.0)	−0.62 (1.4)	0.2

*Note*: Data are median (IQR) (a) or *n* (%) (b) or mean (SD) (c). Fenton growth charts (d). Excludes three infants randomised and withdrawn without informative samples. Medians (IQR) analysed by Mann–Whitney, % by Fisher's exact test and mean (SD) by *t* tests. (e) *S. capitis*, (f) *E. coli*, *K. rhizophila*, (g) *K. pneumonia*, *S. capitis*. No cases of FIP, PHVD, MCO or PVL were seen in either group, * indicates statistical significance at *p* < 0.05.

Abbreviations: CLD, chronic lung disease; *E. coli*, *Escherichia coli*; FIP, focal intestinal perforation; IQR, interquartile range; *K. pneumonia*, *Klebsiella pneumoniae*; *K. rhizophila*, *Kocuria rhizophila*; LOS, late‐onset sepsis; MCO, milk curd obstruction; NEC, necrotising enterocolitis; PBMF, powdered bovine milk‐derived fortification; PHMF, powdered human milk‐derived fortification; PHVD, post‐haemorrhagic hydrocephalus; PMA, post‐menstrual age; PVL, periventricular leukomalacia; ROP, retinopathy of prematurity; *S. capitis*, *Staphylococcus capitis*; SD, standard deviation; SDS, standard deviation score.

### 3.1 Stool sample collection and selection

We collected stool on 120 occasions (each in duplicate); analysis of faecal calprotectin was undertaken in 98 stool samples from 28 infants within the pre‐specified analysis periods. The number of samples analysed at each time point is given (Table S1). The primary outcome was faecal calprotectin on Days 7 and 21, where Day 21 was not available a Day 14 sample was analysed in four infants, included in regression analysis but not in primary outcome analysis. Samples were unavailable due to non‐stooling, transfer, study or product withdrawal before the appropriate time point was reached.

### 3.2 Faecal calprotectin

Faecal calprotectin values by group are given in Table 3. There was no statistical difference in unadjusted faecal calprotectin between PHMF and PBMF at any time point, either for all infants or excluding those with NEC or LOS.

**Table 3:** Unadjusted calprotectin values (mcg/g) at enrolment, Days 7 and 21 by study group, for all infants and excluding those with disease within the first 21 days.

	Study time point			
Group (infant *n*)	Enrolment	Day 7	Day 21	Discharge
Control PBMF^a^ (*n* = 14)	167 (71–681)	303 (123–581)	315 (197–462)	500 (94–1046)
Intervention PHMF (all infants *n* = 14)	219 (54–352)	236 (136–474)	135 (61–417)	425 (260–669)
Intervention PHMF (1 NEC and 2 LOS excluded *n* = 11)	235 (61–349)	234 (102–437)	141 (49–591)	354 (80–606)

*Note*: (a) Control group did not experience any NEC or LOS within the first 21 days. All PBMF vs. PHMF comparisons were nonsignificant at all time points (*p* > 0.05 by Mann–Whitney) Calprotectin values are median (IQR).

Abbreviations: IQR, interquartile range; LOS, late‐onset sepsis; NEC, necrotising enterocolitis; PBMF, powdered bovine milk‐derived fortification; PHMF, powdered human milk‐derived fortification.

For the first 21 days, Spearman correlation identified gestation and day of life as significantly correlated to faecal calprotectin; after adjusting for these, there was no impact of fortifier type on calprotectin. No change was seen when infants with NEC or LOS were removed from this analysis. Using samples from all infants (Days 7, 14, 21 and discharge), Spearman correlation identified only gestation as significantly correlated to calprotectin and adjusting using multiple regression again found no impact on faecal calprotectin of the trial arm (Table 4).

**Table 4:** Impact on faecal calprotectin of key factors.

Population	First 21 days, all infants (*n* = 28)	First 21 days, no LOS or NEC infants (*n* = 25)	All infants, all samples including Day 14 and discharge (*n* = 28)
Factor:	**Spearman correlation:** *R* (95% CI) *p*		
Day of life	−0.37 (−0.59 to −0.09)**	−0.39 (−0.62 to −0.10)**	−0.10 (−0.29 to 0.12)
Day after fortification started	−0.15 (−0.41 to 0.14)	−0.08 (−0.37 to 0.23)	0.13 (−20.08 to 0.33)
Gestation (weeks)	0.31 (0.03–0.55)*	0.36 (0.07–0.6)*	0.32 (0.13–0.50)**
Days fortified	−0.17 (−0.44 to 0.12)	−0.099 (−0.39 to 0.21)	0.11 (−0.1 to 0.31)
PMA	0.06 (−0.2 to 0.34)	0.12 (−0.18 to 0.41)	0.18 (−0.03 to 0.37)
Factor:	**Multiple regression:** Effect estimate (95% CI) *p*		
Trial arm (PHMF vs. PBMF)	1.32 (−165 to 167)	−7.13 (−172 to 158)	−67 (−231 to 96)
Day of life	−6.2 (−13 to 1.1)	−5.7 (−12.9 to 1.53)	2.38 (−2.6 to 7.3)
Gestation (per week)	6.10 (−32 to 45)	19.3 (−18 to 57)	33.14 (−5.6 to 72)

Abbreviations: CI, confidence interval; LOS, late‐onset sepsis; NEC, necrotising enterocolitis; PBMF, powdered bovine milk‐derived fortification; PHMF, powdered human milk‐derived fortification; PMA, post‐menstrual age.

* *p* < 0.05

** *p* < 0.005.

### 3.3 Plasma amino acids

Plasma amino acid concentrations on Days 7 and 21 were analysed in 12 and 13 infants in the PHMF group and 12 and 7 and in the PBMF group, respectively. Infants with missing results did not have blood available for analysis. There were statistically significant differences at Day 7 between groups in glutamate (higher in PHMF), glycine (higher in PHMF) and threonine (lower in PHMF) and at Day 21 in cystine (higher in PHMF), threonine (lower in PHMF) and tyrosine (lower in PHMF). For all except leucine and 1‐methylhistidine, some values for infants in both groups fell outside paediatric reference data from the CALPIER study, used as neonatal reference data is lacking (14%–100% of infants outside range^16^) (Table S2).

## 4 DISCUSSION

This RCT of infants <32 weeks of gestation on EHMD compared the effect of a PHMF to PBMF on gut inflammation using faecal calprotectin. We believe this is the first RCT using powdered forms of both human and bovine‐derived fortifiers, and thus, is important.

The study stopped early due to manufacturer recall and withdrawal of the PHMF due to manufacturing issues. Our power calculation suggested that 36 infants were needed; 28 recruits contributed to our primary analysis. We found no difference in faecal calprotectin within 21 days of fortification for all infants or after excluding those with NEC or LOS. Adjusting for gestation and day of life, both independently associated with faecal calprotectin, did not change this. We did not find statistical differences in fortifier use, tolerance, stooling, NEC, FIP, IVH or MCO between groups, but PHMF infants exhibited slower weight gain, statistically significant at discharge. There was a small (non‐significant) difference in day of life at recruitment between groups, meaning the fortifier started slightly later in the PHMF arm, which may have contributed to this difference. Donor milk use was also more common and used for more days in the PHMF group, although not statistically significant for either measure in the first 21 days after fortification. Many infants in both groups were discharged on exclusive MOM, but where this was not possible, more infants in the PHMF arm were supplemented with donor milk, and in the PBMF, more were supplemented with formula. This may have impacted growth at discharge. Milk type after Day 21 was at the clinician's discretion, but the use of donor milk in the PHMF group may reflect a preference to continue to avoid bovine products given the human fortification.

Plasma amino acid profiles differed between groups, but not consistently, and many infants in both groups had concentrations of amino acids outside our reference range. The potential importance of these differences is unclear, but given the different protein sources and growth differences, they merit further exploration.

We used a biochemical marker of gut inflammation as the primary outcome. Previous data does suggest faecal calprotectin is higher in preterm infants with gut inflammation or feed intolerance: infants with NEC have higher faecal calprotectin levels than healthy infants.^15^, ^17^, ^18^ In meta‐analysis of preterm NEC cases elevated faecal calprotectin had a sensitivity of 0.91 and specificity of 0.93 for NEC suggesting it is a robust marker of gut inflammation when present as part of established disease.^18^ Poor tolerance to enteral feeding, defined as unplanned enteral feed interruption, has also been associated with higher faecal calprotectin,^19^ even in the absence of definitive disease. Previous reports also show diet impacts calprotectin, but relatively small numbers of babies have previously been studied. In unadjusted analysis, faecal calprotectin levels were significantly lower in infants receiving human milk than those receiving formula^19^ and infants receiving bovine protein as either formula or fortifier had significantly elevated faecal calprotectin levels compared to those receiving EHMD, but the analysis was not adjusted for other factors that influence calprotectin such as day of life.^14^ Addition of PBMF to breastmilk has been associated with elevation in faecal calprotectin immediately after starting PBMF.^20^ The lack of reduction in calprotectin in PHMF infants in our study may relate to adjusting for confounding factors, or sampling after an established duration of supplementation rather than immediately (first sample 7 days after first fortification) meaning we may have missed a short‐lived initial PBMF‐mediated calprotectin rise.

Previous studies in EHMD infants comparing HMF to BMF use liquid HMF, displacing MOM, which may negate a beneficial effect of HMF, a problem not seen in our study design where both fortifier types were powdered. A blinded RCT of LHMF compared to PBMF did not identify differences in faecal calprotectin in the first 2 weeks of fortification. Clinical outcomes and growth did not differ.^13^ In contrast in 30 very low birth weight infants Kumbhare et al. identified significantly higher calprotectin levels in infants fortified with LHMF compared to PBMF.^21^ The recent multi‐centre Swedish N‐Forte study randomised infants of 22–28 weeks of gestation to LHMF or PBMF, fortifying on an individualised targeted basis, and not specifying the specific PBMF product.^22^, ^23^ No difference in composite primary outcome of death/stage II or III NEC or LOS nor in key secondary clinical outcomes including weight at 34 weeks of PMA were seen. This lack of slower growth in the LHMF arm may have been the result of targeted fortification which we did not do. A similar study design in 126 infants on a human milk diet, randomised to LHMF or PBMF, showed no differences in time to full feeds, NEC, other key neonatal morbidities, change in weight SDS at 34 weeks of PMA^24^ or body composition.^25^ This study was powered to explore putative benefits of an EHMD through microbial impacts but no differences in overall gut bacterial diversity were seen, although there were effects on specific bacterial taxa previously associated with human milk receipt.^24^

Other studies comparing PBMF and LHMF do not use EHMD and so are not directly comparable to our study.^10^, ^26^ However, meta‐analysis of four RCTs (241 infants) comparing LHMF and PBMF regardless of whether the diet was otherwise EHMD or allowed formula, like ours, identified overall lower weight gain in the HMF group.^27^ The biological mechanism and clinical importance of this difference in weight gain remains uncertain.^28^

A major strength of our study is the powdered nature of both fortifier products, avoiding the potential impact of displacement of a volume of MOM seen in studies that use LHMF. Our study population were high‐risk preterm infants at risk of feed intolerance, growth failure, NEC and LOS, outcomes all previously linked to diet and where the exclusive role of fortifier type within an otherwise EHMD remains to be established. There was no loss to follow up and despite early study termination, the groups were well balanced. We adjusted the calprotectin analysis for day of life and gestation, especially important where fortification began only after reaching full enteral feeds which occurs at variable PMA. Our study is, however, underpowered due to the PHMF becoming unavailable due to manufacturing issues.

## 5 CONCLUSION

Clinical outcomes must be viewed in light of small numbers of infants, but differences in growth still reached significance at discharge, and are in keeping with meta‐analysis of HMF versus BMF.^27^ Although underpowered the lack of clinical impact is consistent with the adequately powered clinical trial by Jensen et al.^23^ and the lack of effect on calprotectin also seen in the O'Connor trial.^13^ Other mechanisms to reduce risks of adverse outcomes in preterm infants from complications that appear to involve feeding or microbiomic mechanisms, and maximising MOM intake deserve further exploration.

## CONFLICT OF INTEREST STATEMENT

Drs Janet Berrington, Nicholas Embleton, and Christopher Stewart report grants to their institutions from Prolacta Biosciences US (Duarte, CA, USA), and grants from Danone Early Life Nutrition (Paris, France). Dr Nicholas Embleton declares lecture honoraria donated to charity from Nestlé Nutrition Institute (La Tour‐de‐Peilz, Switzerland). Dr Christopher Stewart declares lecture honoraria from Nestlé Nutrition Institute (La Tour‐de‐Peilz, Switzerland). The remaining authors declare no conflicts of interest.

## Supporting information

Supporting information.

## Data Availability

Suitably anonymised data may be available on reasonable request.
