# Supplementary material for: Powdered human milk‐derived versus bovine milk‐derived breastmilk fortification: A multi‐centre preterm randomised controlled trial
Source: J Pediatr Gastroenterol Nutr. 2024 Dec 11;80(2):336–44. doi: 10.1002/jpn3.12431 (PMC11788938; doi:10.1002/jpn3.12431)
Supplement: Supplementary file 1 — Supporting information. [file JPN3-80-336-s001.docx]

**Supplementary material**

**Supplementary Figure 1 CONSORT Flow Diagram**

Allocation

Suitable samples for analysis (n = 14)

Lost to follow up (n = 0)

Enrolment

Suitable samples for analysis (n = 14)

Lost to follow up (n = 0)

Allocated to control PBMF (n = 15)

- Received allocated intervention (n = 15)
- No informative samples (n=1)

Analysis

Randomized (n = 31)

Assessed for eligibility (n = 50)

Follow up

Excluded (n = 19)

- Declined to participate (n = 17)
- Not approached (n = 2)

Allocated to intervention PHMF (n = 16)

- Study stopped before intervention commenced (n = 1)
- Clinician lost equipoise (n = 1)
- Received allocated intervention

(n =14)

**Supplementary Table 1** Number of stool samples analysed by group and time point

| Sample details | PBMF (14) | PHMF (14) | Median day of study (IQR) |
| --- | --- | --- | --- |
| Enrolment | 14 | 13 | 0 (0-0) |
| Day seven | 14 | 14 | 7 (7-8) |
| Day 14 | 3 | 1 | 14 (14-14_ |
| Day 21 | 9 | 13 | 21 (21-22) |
| Discharge | 7 | 10 | 29 (37-45) |

**Supplementary Table 2** Plasma amino acid concentrations on day seven and day 21 by study group

| **Amino acid reference intervals (µmol/L)**  **18)** | **Control PBMF**  **Median (IQR) [% outside ref range]** | | **Intervention PHMF**  **Median (IQR) [% outside ref range]** | |
| --- | --- | --- | --- | --- |
|  | **Day 7 (n=12)** | **Day 21 (n=7)** | **Day 7 (n=12)** | **Day 21 (n=13)** |
| Alanine(208– 588)  1 wk to < 19 yrs | 217 (182-352)  [41] | 317 (238-35)  [14] | 317 (238-335)  [17] | 298 (201-390)  [23] |
| α-amino Butyric acid (8-30)1 wk to < 19 yrs | 4 (0-6)  [88] | 7 (3-12)  [50] | 4 (2-5)  [100] | 4 (2-6)  [100] |
| Arginine (47–138)  1 month to < 1 yr | 58 (35-67)  [33] | 70 (43-82)  [28] | 75 (55-84)  [17] | 57 (47-85)  [23] |
| Asparagine (38–91)  0 to < 19 yrs | 72 (58-121)  [17] | 73 (52-117)  [20] | 75 (64-88)  [14] | 69 (65-90)  [15] |
| Aspartate (20–42)  2 wks to < 19 yrs | 6 (6-14)  [77] | 6 (5-28)  [80] | 8 (7-39)  [72] | 7 (6-13)  [86] |
| Citrulline (9 – 44)  0 to < 1 yr | 19 (16-26)  [0] | 20 (17-31)  [14] | 19 (15-23)  [0] | 23 (18-16)  [8] |
| Cystine(3 – 20)  2 wks to < 8 yrs | 25 (23-30)  [88] | 24 (23-28)*  [100] | 31 (26-35)  [100] | 32 (25-40)*  [100] |
| Glutamate (74–266)  2 wks to < 1 yr | 50 (41-75)*  [69] | 64 (45-104)  [57] | 92 (54-136)*  [31] | 84 (50-110)  [46] |
| Glutamine (332–789)  1 wk to < 1 yr | 370 (307-572)  [17] | 449 (335-622)  [14] | 443 (335-622)  [34] | 458 (323-570)  [15] |
| Glycine (196 – 398)  2 wks to < 13 yrs | 184 (173-193)*  [83] | 190 (187-226)  [57] | 215 (191-317)*  [34] | 187 (124-224)  [59] |
| Histidine (65-113) 2 wks to < 19 yrs | 83 (59-120)  [58] | 90 (57-180)  [71] | 114 (85-144)  [50] | 110 (71-155)  [62] |
| Isoleucine (30 – 113)  2 wks to < 1 yr | 62 (49-82)  [17] | 58 (51-62)  [0] | 54 (46-66)  [0] | 53 (43-64)  [8] |
| Leucine (55 – 188)  1 wk to < 1 yr | 90 (75-131)  [0] | 89 (85-116)  [0] | 103 (79-124)  [0] | 97 (76-119)  [0] |
| Lysine (102 – 259)  2 wks to < 19 yrs | 185 (147-214)  [33] | 159 (73-195)  [43] | 137 (63-213)  [58] | 72 (62 – 207)  [59] |
| Methionine (13–44)  0 to < 19 yrs | 26 (21-33)  [17] | 28 (22-30)  [0] | 24 (18-31)  [8] | 25 (19-34)  [0] |
| Ornithine (40–132)  2 wks to < 1 yr | 99 (85-125)  [8] | 99 (95-132)  [14] | 133 (95-200)  [42] | 105 (79-137)  [23] |
| Phenylalanine  (52–116)2wks to<1 yr | 46 (41-55)  [42] | 50 (43-53)  [71] | 50 (43-59)  [58] | 40 (37-56)  [70] |
| Proline (127–292)  0 to < 1 yr | 180 (142-219)  [25] | 191 (129-229)  [14] | 168 (139-221)  [0] | 175 (142-206)  [15] |
| Serine (112 – 216)  2 wks to < 19 yrs | 128 (113-147)  [25] | 141 (120-159)  [28] | 168 (125-263)  [50] | 124 (101-155)  [15] |
| Taurine (55 – 204)  2 wks to < 19 yrs | 84 (51-104)  [25] | 94 (59-136)  [14] | 100 (74-152)  [8] | 74 (50-93)  [31] |
| Threonine (27–187)  2 wks to < 19 yrs | 251 (190-308)*  [75] | 210 (171-241)*  [57] | 134 (109-218)*  [42] | 150 (97-168)*  [15] |
| Tyrosine (34–151)  2 wks to < 19 yrs | 108 (78-161)  [33] | 131 (114-189)*  [14] | 97 (65-142)  [8] | 101 (68-121)*  [0] |
| Valine (128 – 361)  2 wks to < 13 yrs | 137 (117-183)  [17] | 142 (122-148)  [29] | 131 (106-162)  [42] | 133 (102-161)  [38] |
| 1methylhistidine  (1-30) 2 wks to <19yrs | 4 (4-7)  [0] | 4 (4-10)  [0] | 6 (3-8)  [0] | 4 (4-8)  [0] |
| 3methylhistidine  (4-18) 1 wk to < 1 yr | 4 (2-6)  [44] | 3 (2-4)  [80] | 3 (3-4)  [58] | 3 (2-3)  [86] |

* p<0.05 PBMF vs PHMF by Mann Whitney

**Protocol**

Breastmilk fortification in preterm infants: a randomised controlled trial of two nutritionally equivalent fortifiers (PUFFIN – Preterm milk Fortification in Neonates)

**Chief Investigator**: Dr Janet Berrington, Ward 35, Royal Victoria Infirmary, Newcastle upon Tyne NE1 4LP, UK

**Sponsor**: Newcastle Hospitals NHS Foundation Trust

**IRAS Project ID**: 293189

**Sponsor Protocol number**: NuTH 09876

**Trial registration number**: ISRCTN 22484792

**REC reference number**: 21/YH/0224

**SIGNATURE PAGE**

**Chief Investigator**

**Name: Janet Berrington janet.berrington1@nhs.net**

**Title: Consultant Neonatal Paediatrician**

**Signature:**
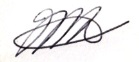
 **Date 09/02/2022**

**Sponsor Representative**

**Name:**

**Title: Research and Development Manager Newcastle Hospital NHS Trust**

**
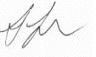
**

**Signature: Date: 09/02/2022**

**Investigator team**

Dr Mark Johnson Consultant Neonatal Paediatrician, University Hospital Southampton

Dr Shalabh Garg Consultant Neonatal Paediatrician, James Cook University Hospital

Dr Chris Stewart Faculty Research fellow, Newcastle University

Dr Chris Lamb Senior Lecturer, Newcastle University

Prof Nicholas Embleton Consultant Neonatal Paediatrician, Newcastle Hospitals

Contents

[Trial Steering Committee (TSC) 7](#_Toc57141621)

[Study proposal summary 7](#_Toc57141622)

[Introduction 9](#_Toc57141623)

[Proposed study to compare two nutritionally equivalent fortifiers 10](#_Toc57141624)

[Inclusion and Exclusion criteria 10](#_Toc57141625)

[Primary and key secondary outcomes 10](#_Toc57141626)

[Additional secondary outcomes – growth and neonatal outcomes 10](#_Toc57141627)

[Sample size and Power 11](#_Toc57141628)

[Funding 12](#_Toc57141629)

[Commercial involvement 12](#_Toc57141630)

[Trial registration, Ethics, HRA and risk burden 12](#_Toc57141631)

[Patient Public Involvement and importance to the NHS 13](#_Toc57141632)

[Experience of research team members 14](#_Toc57141633)

[References 16](#_Toc57141634)

[Appendix 1: data items 17](#_Toc57141635)

[Appendix 2: Patient Information Sheet (PIS) 19](#_Toc57141636)

[Appendix 3: Consent form 23](#_Toc57141637)

Appendix 4: Suggested vitamin and mineral supplementations

# Trial Steering Committee (TSC)

| **Name** | **Role** |
| --- | --- |
| TBC | Chair / Independent member |
| Dr Janet Berrington | Chief Investigator |
| Dr Mark Johnson | Co-investigator |
| Dr Shalabh Garg | Co-investigator |
| Dr Nicholas Embleton | Co-investigator |
| TBC | parent |

# Study summary

This is a protocol for an Investigator Initiated trial, commercially part-funded, non-blinded, randomised controlled trial comparing two nutritionally equivalent methods of breastmilk fortification in healthy preterm infants. The dietary regimes involve fully supporting mothers to provide their own expressed breast milk and using donor human milk to make up shortfall in breastmilk supply. The primary outcome is the level of inflammatory markers in the stool. Parents of infants on neonatal units (Newcastle upon Tyne, Middlesbrough and Southampton) will be approached by clinical team members, and infants will be enrolled after signed informed consent. The study starts when infants are stable and tolerating full milk feeds and finishes prior to hospital discharge and will last for around 3-6 weeks per baby on average.

| Title (Acronym) | **PUFFIN – Preterm Milk Fortification in Neonates** |
| --- | --- |
| Study centres | Ward 35, Royal Victoria Infirmary, Newcastle upon Tyne (lead site)  James Cook University Hospital, Middlesbrough  University Hospital Southampton |
| Study objectives | To compare two comparable methods of fortifying mother’s own breastmilk and/or donor human milk |
| Study design | Randomised, minimised, open label, controlled trial |
| Study population | Inclusion criteria   - Stable preterm infants born <32 completed weeks of gestation or <1500g birthweight tolerating full milk feeds for at least 24 hours - Written informed consent from parents - Maternal intention to continue providing breastmilk - Agreement to use breast milk fortifier   Exclusion criteria   - Parents unwilling to consent - Exposure to formula milk or breastmilk fortifiers before randomisation - Known congenital anomaly affecting the GI tract (gut) - Previous gut surgery or diagnosis of necrotising enterocolitis (NEC) |
| Interventions | Standard commercially available breastmilk fortifier derived from bovine source added to breast milk (control) compared to commercially available fortifier derived from human milk (intervention) |
| Duration | From full milk feeds until fortifier no longer required, or 36 weeks corrected gestation (whichever is earlier) |
| Target number of patients | 36 infants with complete sampling (18 infants per trial arm): recruitment of up to 50 infants may be needed to achieve this |
| Randomisation | Minimisation using gestation (<28 weeks yes/no) and hospital site. Twins and triplets will be allocated to same study arm. Secured, password protected web-based randomisation using minimisation algorithm ([www.sealedenvelope.com](http://www.sealedenvelope.com) or similar) |
| Primary outcomes | Stool inflammatory marker (calprotectin) |
| Secondary outcomes | Fecal cytokine levels  16s gut microbiota profile  sIgA in stool  Urinary inflammatory markers (iFABP and similar)  Feed tolerance and growth-related outcomes  Neonatal morbidities and clinical outcomes |
| Duration of trial intervention | 36 weeks postmenstrual age or when fortifier no longer required (whichever earlier) |
| Duration of study | Recruitment period: 12 months  Total trial duration: 15 months |
| End of Trial | Discharge from neonatal unit |
| Safety assessments | Routine assessments until discharge from Neonatal Unit  Safety tracking during hospitalisation |

## Introduction

Around 10% of all babies are born premature, but whilst the majority do not require specialist medical treatment, those born very preterm (VPT, <32 weeks’ gestation) require prolonged hospital stay including intensive care. Survival in these VPT infants has increased dramatically in recent years, but death is still common (~10% overall) as are the consequences of life-long physical and cognitive impairment.[1] In the UK around 10,000 VPT infants are born every year, representing an annual cost to the NHS of ~£3 billion.[2] The commonest cause of death or serious illness in preterm infants after the first few days are gut complications such as necrotising enterocolitis (NEC) or septicaemia.[3] Morbidity and mortality is lower in infants who receive mother’s own expressed breastmilk (MOM) and around 90% of mother’s provide at least some breastmilk. When there is a shortfall in MOM supply, an alternative milk is needed, which is either formula milk, or pasteurised, donated, human milk (DHM).[4,5]

A further challenge arises because human breast milk alone will not meet the nutrient requirements of most VPT infants.[6] To meet the higher nutrient requirements of VPT infants, commercially produced breast-milk-fortifiers are milk-powdered products added to mothers’ own milk or donor human milk.[6] These increase the energy and protein density of the milk (along with additional micronutrients, vitamins etc.) and have been routinely used in neonatal units for the last 20-25 years. Most currently used fortifiers are derived from bovine sources (cow milk fortifier, CMF) and are therefore a similar product to cow’s milk infant formula. Whilst routine use of CMF appears to be very safe, there are concerns that using bovine products may increase the risk of allergy, cow milk protein intolerance or other inflammatory problems in preterm infants,[7] however without using CMF most preterm infants will not grow adequately. Poor growth in VPT infants is an important concern because it is strongly linked to worse longer-term cognitive outcomes and may dissuade the mother from continuing to provide breastmilk.[8]

Human milk-derived fortifiers (using donated human breastmilk) have been developed over the last few years and have been widely used in the US for more than 10 years.[9] These have the advantage of avoiding exposure to bovine proteins, and may provide a more optimal balance of nutrients (especially fatty acids and human milk oligosaccharides), but have only been available as a frozen concentrated liquid which makes logistics and transport more complex and these products are consequently more expensive than standard bovine sourced CMF.[9] More recently, human-milk-derived fortifiers have been developed as powders, meaning they are available at a lower cost and do not displace a volume of mother’s own breastmilk (a problem of using a liquid based fortifier, which can be significant at higher doses). Whilst even powdered HMF will be much more expensive than CMF, the additional expense may be justified if these improve longer term infant outcomes. As an initial study, we want to determine if there are any measurable differences in levels of gut inflammation between breastmilk fortifiers derived from cow milk sources (cow milk based fortifier (CMBF), standard, control) to fortifiers derived from human milk (human milk based fortifier (HMBF), intervention).

## Proposed study to compare two nutritionally equivalent fortifiers

The Puffin study is designed to compare two nutritionally equivalent dietary approaches, by comparing the use of standard cow milk fortifiers (CMBF, control) to that of using human milk fortifier (HMBF, intervention).

- **Setting**: Three tertiary level NICUs
- **Population**: preterm infants <32 weeks or <1500g tolerating milk feeds
- **Control**: use of CMBF from full feeds (tolerating 150mls/kg/day for 1-2 days)
- **Intervention**: use of HMBF from full feeds
- **Primary outcome**: stool inflammatory markers (calprotectin) after 3 weeks exposure
- **Study design**: non-blinded randomised controlled trial
- **Timeframe**: until 36 weeks corrected gestation or when fortifier no longer required whichever is earlier

### Inclusion and Exclusion criteria

To be included, infants must have been born at <32 weeks gestation and/or <1500g and be tolerating full milk feeds for at least 24 hours. Infants who have any congenital abnormalities that affect feeding, or who have had gut surgery or a diagnosis of necrotising enterocolitis (NEC), will be excluded. In addition, infants who have been exposed to formula milk or breastmilk fortifiers before randomisation will be excluded.

Randomisation

Infants will be randomised using minimisation incorporating the following variables: hospital site, gestation (<28 weeks), and multiple birth status; this will be held and coordinated by the Newcastle research team using an internet based secured randomisation system. Contact and allocation will be by password protected internet access. Infants will be allocated to either standard fortification (control) or human based fortification (intervention). Multiples (twins and higher order) will be randomised to the same trial arm.

### Primary and key secondary outcomes

We will determine stool inflammatory profile by measuring fecal calprotectin after 1 and 3 weeks of exposure (primary outcome) and a panel of fecal cytokines using a multiplex assay (<https://www.mesoscale.com/products/v-plex-human-biomarker-54-plex-kit-k15248d/>) similar to that used in recent studies exploring the impact of probiotics in VPT infants.[10] We will also determine fecal microbiota proportions using 16s RNA next generation sequencing as used in our previous studies[11] and secretory IgA (sIgA) levels in stool.

### Additional secondary outcomes – growth and neonatal outcomes

We will collect a range of relevant secondary outcomes:

1. Growth – weekly weight, length and head circumference, absolute changes (g/kg/day and mm/week) and change in standard deviation score based on UK reference data from study enrolment until 36wca and/or discharge
2. Episodes of NEC, sepsis - confirmed and clinically suspected according to existing nationally agreed case definitions
3. Chronic lung disease, Retinopathy of prematurity (ROP), Intraventricular haemorrhage, Cystic PVL etc.
4. Days of intensive, high and low dependency care; age at discharge, total length of stay
5. Age at starting fortifier; tolerance, days not received due to intolerance, duration of breastfeeding; type of feeding at discharge (direct breast feeding, tube feeding etc.) etc.
6. Blood/urine work, serum biochemistry and other inflammatory and immune markers (blood and urine): hsCRP, lymphocyte subsets, plasma amino acids (PAA), where samples and funding allow

|  | Enrolment (baseline) | Day 7 | Day 14 | Day 21 | 36w or discharge | Total per infant |
| --- | --- | --- | --- | --- | --- | --- |
| Growth | x | x | x | x | x | 5 |
| Calprotectin | x | x | x | x |  | 4 |
| Stool cytokines |  | x |  | x |  | 2 |
| Stool 16s RNA | x | x |  | x |  | 3 |
| Stool sIgA | x | x |  | x |  | 3 |
| Urine inflammatory/ oxidative measures | x | x | x | x | x | 5 |
| Feeding tolerance etc. | x | x | x | x | x | 5 |
| Targeted bloodwork (PAA etc) |  | x |  | x |  | 2 |
| Clinical outcomes / safety | x | x | x | x | x | 5 |

### Sample size and Power

We hypothesise that use of a HMF will result in lower levels of fecal calprotectin, and a panel of fecal cytokines and that this will also be associated with greater abundance of Bifidobacteria using 16s gut microbiota analysis. Calprotectin levels in preterm infants vary depending on the presence of neonatal disease but are typically between 4 and 6±2mg/dL[12]. To determine a 50% reduction in fecal calprotectin levels we will study the primary outcome assessment in 36 infants using 5% significance and power of 80%. Allowing for dropouts and other reasons we aim to recruit between 40-50 infants across three NICU sites.

## Funding

We have acquired funding support from NeoKare to cover the costs of study delivery, fecal assays and 16s RNA analysis (total costs provided £50,000) and supply of HMF product free of charge. The remaining non-consumable costs will be limited because there is no other change to clinical practice, and we will collect electronic clinical data extracted from e-record and Badger neonatal database which will be inputted into an excel worksheet. Trial recruitment will be conducted by a GCP member of neonatal research staff supported by the neonatal research nurse. Costs additional to £50,000 (e.g urinary assays etc) will be met out of existing departmental research funds.

## Commercial involvement

NeoKare will play no role in any aspect of trial conduct, and all data will remain the property of NHS Hospitals who will undertake data collection and analysis along with established collaborators at Newcastle University. Newcastle lead site will develop a contract with NeoKare to allow them sight of any data prior to publication but the right to publish study findings will rest entirely with the research team. NeoKare will retain full IP of their product.

## Trial registration, Ethics, HRA and risk burden

The trial will be an Investigator Initiated trial registered on ISRCTN prior to opening sponsored by Newcastle Hospitals NHS Foundation Trust. We will apply for HRA and REC approvals and apply for adoption onto the NIHR portfolio. The trial is an open-label study of two comparable diets and is not a CTIMP. There are no specific ethical issues with this trial, but we recognise the additional burden placed on parents by being asked to consider trial enrolment. There are no specific safety or risk issues as part of the trial *per se* as all milk and products (donor milk, donor milk fortifier, cow milk-based fortifier, mother’s own milk) are already given routinely to babies. No additional interventions or tests are required for infants to join the trial, but we will seek permission to collect an extra sample of blood at the same time as venepuncture is required for any clinical reasons on two occasions. Parents can choose to decline the additional targeted blood samples but still participate in the trial as all other tests are non-invasive. The trial is fully compliant with national recommendations and Baby Friendly Initiative (BFI) part of UNICEF.

Patient withdrawal

Temporary discontinuation of the intervention is at the discretion of the attending clinician.

Permanent discontinuation is possible at parental request at any point, or after transfer to a non-participating site, and may be accompanied by permission to continue to collect data or not. Data already obtained will be included in all analyses.

Trial product

Standard cows milk based fortifiers in current use in each recruiting unit will be utilised by each site in the control arm, and be subject to standard vitamin and mineral supplementation in use in that unit, or as suggested in Appendix 4 if no current guidance exists. Infants randomised to human based fortifier will use NeoKare Maternal Milk Fortifer (MMF) and the vitamin and mineral supplementation outlined in Appendix 4. In all trial arms supplementation should then be adjusted with routinely measured blood results. Infants in both trial arms where insufficient maternal milk is available should receive donor human milk until at least D21 of the study (primary study end point). Thereafter if there is shortfall in maternal milk supply supplementation with donor milk or formula is at the discretion of the attending clinicians.

Adverse events

As this is not a clinical trial of a medicinal product and the intervention, and its delivery, are considered safe and already available to purchase we do not anticipate any adverse reactions due to the trial intervention (use of donor breast milk based fortifier). However, this is a very high risk patient group and some infants may develop serious complications e.g. sepsis or necrotising enterocolitis and some may die. We will record all serious adverse events (SAE). Serious adverse events that may occur include:

• Outcomes recorded and reported as part of the study: necrotising enterocolitis (NEC), sepsis, intraventricular haemorrhage, periventricular leukomalacia, retinopathy of prematurity, chronic lung disease, feeding intolerance (number of days when nil by mouth i.e. no enteral intake or fortification pasued), and death.

• Outcomes that commonly occur in this population but that will not be specifically recorded or reported as part of this study include: abnormal electrolytes, bone chemistry, liver functions tests or other routine biochemistry or haematology; transfusion of blood or related products; complications related to use of intravenous catheters (e.g. misplacement or malfunction); presence of and/or treatment for a patent ductus arteriosus; requirement for surgery and/or provision of an intestinal or airway stoma, intraventricular shunt; vomiting, gastro-oesophageal reflux, constipation, diarrhoea, skin or nappy rash.

Data queries, missing data

Where infants are transferred to other hospitals before the end of the study (36 weeks corrected age) the investigators will make contact with that hospital and request to collect data. The data collected will already form part of the infants’ medical record and can be extracted from clinical records or clinical databases e.g. the National Neonatal Research Database that holds data from the Badger system. The data will include feeding information (e.g. type of milk and supplements), growth (e.g. weight, length and head circumference), any important medical outcomes (e.g. infection episodes, requirement for respiratory support) and duration of hospital stay.

## Patient Public Involvement and importance to the NHS

We have discussed similar studies of milk feeding with parents on the neonatal unit who tell us that the risks and complication associated with feeding preterm infants are very important issues. In addition, the James Lind alliance (JLA) priority setting [www.jla.nihr.ac.uk/priority-setting-partnerships/preterm-birth/top-10-priorities/](http://www.jla.nihr.ac.uk/priority-setting-partnerships/preterm-birth/top-10-priorities/) identifies feeding and nutrition as very important.

This trial is of major relevance to the NHS and studies interventions and outcomes highlighted in a recent top research priority setting in preterm infants.[13] Many parents wish to breastfeed or provide expressed breastmilk, and some parents are concerned about using cow-milk based products. Breastfeeding is a major national priority and is an intervention that has potential to benefit marginalised and poorly resourced groups. Our study focuses on gut inflammation which may be a proxy marker for a higher risk of NEC. NEC is a devastating condition, and half of the infants requiring surgery die or have long-term serious disability. HMBF products could represent an important cost for the NHS, but the total costs of these products even when given to all infants on a large NICU will still be substantially less than the costs associated with a single episode of surgical NEC. We will invite a parent or parent representative to join the TSC. We will liaise with Tiny Lives charity and Bliss UK to consider the implications of our trial findings and disseminate the results.

## Experience of research team members

We are an experienced research team, having recruited more than 1000 preterm infants to trials and studies in the last 10 years, including qualitative exploration of sensitive issue in parents whose babies did not survive. We are recognised internationally for our work on feeding and nutrition, and associated complications such as necrotising enterocolitis. We have worked closely with parents on all our studies, and conducted qualitative studies with parents who are approached to join more than one neonatal research study[14].

Trial organisational structure and responsibilities

Trial sponsor

The institution employing the chief investigator (Newcastle Hospitals NHS Foundation Trust) will act as sponsor

Trial steering committee

A Trial Steering Committee (TSC) will be established to oversee the conduct of the study. It is anticipated that the TSC will comprise the lead investigators, an independent chair and a user representative. The TSC will meet twice during the course of the study, once shortly after the start of the study and then annually or as required throughout the course of the study.

Data monitoring and ethics committee

This is a mechanistic study in which trial outcomes will not be analysed until study end. In addition, the dietary regimens used are un-blinded. We therefore do not plan to have a Data Monitoring and Ethics Committee

Trial management

The trial will be reviewed by the trial management group (TMG) at the end of an internal pilot phase (n=5 per trial arm) and key data reviewed. The TMG will consist of the key investigators JB,SG,MJ along with trial managers, and research nurses, and will meet by teleconference months, with more frequent meetings if needed in the set up and initial period of recruitment.

Trial registration

The trial will be registered on the International Standard Randomised Clinical Trial Number registry.

Trial sites

Three large tertiary neonatal units with considerable experience of intervention trials in high risk neonates: Royal Victoria Infirmary Newcastle, James Cook University Hospital Middlesbrough, and University Hospital Southampton.

Investigator responsibilities

Investigators will be responsible for ensuring that institutional (site specific) approval has been obtained as well as Agreements signed off by their Institution prior to the start of the study. Investigators are required to ensure compliance to the Clinical Trial Protocol, Investigators File and any other study instructions as required by the Sponsor or its representatives. Investigators are required to ensure the accuracy of the trial data according to the instructions provided. Investigators are required to allow access to study documentation or source data on request for monitoring visits and audits performed by, the Sponsor or any regulatory authorities. The Investigator may appoint co-investigators to assist with conduct of the study locally. All co-investigators must be listed as members of the research team and appropriately trained. The Investigator has overall responsibility for ensuring the conduct of the study locally.

Publication policy and dissemination of results

The results from the trial will be submitted for publication irrespective of the outcome. The Trial Steering Committee will be responsible for approval of the manuscripts prior to submission for publication. At the end of the study, infants’ parents will be able to request a copy of the results of the study from the investigator at that site.

Authorship of presentations and reports related to the study will be in the name of the collaborative group.

There will be no funder or sponsor role in data analysis, presentation or publication. Newcastle Hospitals NHS Foundation Trust will act as sponsor and will require access to study resources and documents in order to provide oversight of governance issues relating to trial conduct. Anonymised, summary data will be provided in publications and may be additionally shared with the funder if not included in published outputs. Individual patient data will be held according to current NHS permissions to enable their use in any future meta-analysis.

# References

1 March of Dimes, PMNCH, Save the Children W. Born too soon. *Born Too Soon, Glob Action Rep Preterm Birth Eds CP Howson, MV Kinney, JE Lawn World Heal Organ Geneva* 2012;**13**:1–126. doi:http://whqlibdoc.who.int/publications/2012/9789241503433_eng.pdf

2 Khan KA, Petrou S, Dritsaki M, *et al.* Economic costs associated with moderate and late preterm birth: A prospective population-based study. *BJOG An Int J Obstet Gynaecol* 2015;**122**:1495–505. doi:10.1111/1471-0528.13515

3 Berrington JE, Hearn RI, Bythell M, *et al.* Deaths in preterm infants: Changing pathology over 2 decades. *J Pediatr* 2012;**160**:49-53.e1. doi:10.1016/j.jpeds.2011.06.046

4 Menon G, Williams TC. Human milk for preterm infants: why, what, when and how? *Arch Dis Child Fetal Neonatal Ed* 2013;**98**:F559-62. doi:10.1136/archdischild-2012-303582

5 Renfrew MJ, Craig D, Dyson L, *et al.* Breastfeeding promotion for infants in neonatal units: a systematic review and economic analysis. *Health Technol Assess* 2009;**13**:1–146, iii–iv. doi:10.3310/hta13400

6 Young L, Embleton ND, McCormick FM, *et al.* Multinutrient fortification of human breast milk for preterm infants following hospital discharge. *Cochrane Database Syst Rev* 2013;**2**:CD004866. doi:10.1002/14651858.CD004866.pub4

7 Coviello C, Rodriquez DC, Cecchi S, *et al.* Different clinical manifestation of cow’s milk allergy in two preterm twins newborns. *J Matern Neonatal Med* 2012;**25**:132–3. doi:10.3109/14767058.2012.663171

8 Isaacs EB, Morley R, Lucas A. Early Diet and General Cognitive Outcome at Adolescence in Children Born at or Below 30 Weeks Gestation. *J Pediatr* 2009;**155**:229–34. doi:10.1016/j.jpeds.2009.02.030

9 Sullivan S, Schanler RJ, Kim JH, *et al.* An exclusively human milk-based diet is associated with a lower rate of necrotizing enterocolitis than a diet of human milk and bovine milk-based products. *J Pediatr* 2010;**156**:562-7.e1. doi:10.1016/j.jpeds.2009.10.040

10 Henrick BM, Chew S, Casaburi G, *et al.* Colonization by B. infantis EVC001 modulates enteric inflammation in exclusively breastfed infants. *Pediatr Res* 2019;**86**:749–57. doi:10.1038/s41390-019-0533-2

11 Zalewski S, Stewart CJ, Embleton ND, *et al.* Brief guide to the analysis, interpretation and presentation of microbiota data. *Arch Dis Child Educ Pract Ed* Published Online First: 2017. doi:10.1136/archdischild-2017-313838

12 Yoon JM, Park JY, Ko KO, *et al.* Fecal calprotectin concentration in neonatal necrotizing enterocolitis. *Korean J Pediatr* 2014;**57**:351–6. doi:10.3345/kjp.2014.57.8.351

13 Oliver S, Uhm S, Duley L, *et al.* Top research priorities for preterm birth: Results of a prioritisation partnership between people affected by preterm birth and healthcare professionals. *BMC Pregnancy Childbirth* 2019;**19**:1–10. doi:10.1186/s12884-019-2654-3

14 Richards J, Rankin J, Juszczak E, *et al.* Parental experiences of being approached to join multiple neonatal clinical trials: qualitative study (PARENT). *Arch Dis Child - Fetal Neonatal Ed* 2020;:fetalneonatal-2020-319031. doi:10.1136/archdischild-2020-319031

# Appendix 1: data items and definitions

At randomisation

• Date and time of birth

• Sex

• Confirmation of eligibility (full eligibility check)

• Date of parental consent

• Name of person taking consent

• Gestational age (in weeks and days)

• Recruiting hospital

• Study allocation

Baseline evaluation

• Birth weight (kg)

• Birth length (cm)

• Head circumference (cm)

• Ethnicity (NHS Ethnicity)

• Mode of delivery

• Use of antenatal steroids

• Date and time first trial intervention administered

Daily evaluations

• Record nutritional intake (type of feed, fortification and type)

- If fortification paused, reason

• If infant is nil by mouth and reason

• Check for SAE

• Record of withdrawal information if relevant

Weekly evaluations

• Weight (kg)

• Length (cm)

• Head circumference (cm)

End of study evaluation

• Weight (kg)

• Length (cm)

• Head circumference (cm)

We will use standard definitions of disease and outcomes refined and validated in our previous studies to collect data including

- Standardized forms for: NEC/gut complications; sepsis

# Appendix 2:

# Patient Information Sheet (PIS)

# Version 2 16/08/2021

**Study name**

# Breastmilk fortification in preterm infants: a randomised controlled trial of two nutritionally equivalent fortifiers (PUFFIN – Preterm milk Fortification in Neonates)

We would like to invite you and your baby to participate in the PUFFIN study (Preterm Milk Fortification in Neonates study). Please read this information carefully; do ask us if you have any questions.

**What is the purpose of this study?**

The purpose of the Puffin study is to find out if there are differences in gut inflammation in preterm (premature) babies that receive either standard breast milk fortifier (made from cow’s milk) or a new fortifier made from donated human milk. These fortifiers are powders that are added to breastmilk to increase the amount of protein and energy and to improve growth. The information from this study will add to information from other studies to improve understanding of the best way to feed preterm babies.

**Background information**

Breastmilk is associated with better outcomes in preterm babies including less gastrointestinal (digestive) and breathing problems. The neonatal staff will do all they can to help you express enough milk for your baby. However, breastmilk needs to have extra protein and energy (and other vitamins) added in order that preterm babies grow effectively. This extra protein and energy is provided as a powdered ‘breastmilk fortifier’ which dissolves in breastmilk. For the last 25 years we have used breastmilk fortifiers made from cow’s milk (similar to infant formula milk) but now fortifiers are available that have been made from donated human breastmilk. These so-called human milk based fortifiers may have a more appropriate mixture of fats and other components for preterm babies, but are much more expensive.

**Why has my baby been chosen?**

Your baby is eligible for this study because s/he was born at < 32 weeks’ gestation or <1500g birthweight, is tolerating full milk feeds and you intend to continue to provide breast milk, and have agreed that we can give your baby breastmilk fortifiers as is standard practice on the neonatal unit. Your baby cannot take part if you are unwilling to consent, they have already been exposed to formula milk or breast milk fortifier prior to randomisation, they have a known congenital anomaly affecting the GI tract (gut) or they have had previous gut surgery or a diagnosis of necrotising enterocolitis (NEC).

**Does my baby have to take part?**

No, it is entirely up to you to decide whether you wish your baby to take part. If you do decide to take part you will be given this information sheet to keep and be asked to sign a consent form, a copy of which will be given to you. If you decide not to take part this will not affect the care that your baby receives.

**What if I change my mind after agreeing for my baby to take part?**

If you decide to take part, you are free to withdraw your baby from the study at any time and without giving a reason. Unless you ask us not to, information collected up to that point will be included in the study.

**What are the differences in the care my baby will receive depending on whether s/he participates in the study?**

The only difference between your baby’s care and that of a baby not in the study is there is a 50:50 (equal) chance of getting the standard cows-milk based fortifier and a 50:50 (equal) chance of getting the new, human milk-based fortifier. Babies who join the study have an equal chance of being in either group. This is done through a process called randomisation where information about your baby (e.g. weeks’ gestation, which neonatal unit they are in) are entered into a computer which randomly allocates your baby to one of the fortifiers. The research team do not know which diet your baby will receive beforehand, and cannot choose. We will also ask your permission to collect stool and urine samples. We hope that around 40 - 50 babies will join the study. We will only analyse the samples once all the babies have finished the studies. This means the results of the study will not change the treatment your baby receives. With your permission we will inform your GP that your baby is taking part.

**What will happen to my baby if s/he takes part?**

We aim to give your baby a breast milk fortifier when your baby is receiving around 150 ml/kg per day of milk. We will continue the fortifier until around 35-36 weeks’ gestation when most babies are getting ready to go home. We collect information about how your baby feeds and grows from the hospital records. We will collect stool and urine samples from your baby’s nappy. We will also ask your permission to collect an extra sample of blood (less than half a teaspoon) on two occasions when the doctors need to take blood for some other reason. This means we will not use an extra needle to collect that blood sample. If you have more than one baby (twins, triplets etc) they will be allocated to the same study intervention.

**What will happen if my baby does not join the study?**

If you choose not to participate, your baby will receive standard care which includes standard cows-milk based breastmilk fortifiers designed for preterm infants.

**What are the possible disadvantages and risks of taking part?**

We do not think there are any risks to taking part in the study. As with all studies, Newcastle Hospitals NHS Foundation Trust that is sponsoring this study holds insurance policies which apply to this study. In the unlikely event of your baby experiencing harm or injury as a result of taking part in the study, you may be eligible to claim compensation without having to prove fault. This does not affect your legal rights to seek compensation.

If your baby is harmed due to someone’s negligence, then you may have grounds for a legal action. Regardless of this, if you wish to complain, or have any concerns about any aspect of the way you have been treated during the course of this study you should inform a local investigator. Normal NHS complaints mechanisms are also available to you.

**What are the possible benefits of taking part?**

We do not know whether individual babies will benefit from taking part, but we hope the study results will help improve the way in which preterm babies are fed. The results may also help inform future decisions about the uses, costs and possible benefits of different products.

**What happens when the research study stops?**

Your baby’s involvement in the study will end when they are discharged from the neonatal unit. We will let you know how you can find out about the results of the study.

**What if relevant new information becomes available?**

Sometimes new information about the treatments being studied becomes available. If this happens, a member of the research team will inform you and discuss any implications.

**Will my baby taking part in this study be kept confidential?**

All information obtained about your baby during the research will be kept strictly confidential. Study information may be looked at by authorised people (audit) to check that it is being carried out correctly, all of whom have a duty of confidentiality to your baby. All data will be handled according to the General Data Protection Regulations (GDPR) 2018.

**What will happen to the results of the research study?**

The results of this study will be presented in scientific journals, and at scientific meetings, submitted to NHS regulatory authorities, and reported on relevant websites such as ClinicalStudyResults.org and www.clinicaltrials.gov.

**Who is organising and funding the research?**

The study has been organised by doctors in Newcastle, Middlesbrough and Southampton. The study is funded in part by the company who are making the new donated human milk fortifier called NeoKare. NeoKare are paying for some of the tests and are providing the hospital with fortifier free of charge. The remainder of the costs will be funded by the Newcastle neonatal team. NeoKare will not be able to see any information about your baby without full anonymisation, and the hospital will take full responsibility for analysing the data.

**Who has reviewed the study?**

All research studies in the NHS are reviewed by a Research Ethics Committee, an independent group of people with responsibility to the Health Research Authority to protect your baby’s rights, safety, wellbeing and dignity. This study has been approved by the XXXXXXXXXXXX *Research Ethics Committee.*

If you want to raise your concerns with someone not involved in your care, you can contact the Patient Advise and Liaison Service (PALS).  This service is confidential and can be contacted on Freephone: 0800 032 0202. Alternatively, if you wish to make a formal complaint you can contact the Patient Relations Department through any of the details below:

Telephone:  0191 223 1382 or 0191 223 1454

Email:   [patient.relations@nuth.nhs.uk](mailto:patient.relations@nuth.nhs.uk)

Address: Patient Relations Department, The Newcastle upon Tyne Hospitals NHS Foundation Trust, The Freeman Hospital, Newcastle upon Tyne, NE7 7DN

Further information and contact details

Please ask us about anything that is not clear, now or at any time during this study. Please speak to the doctor or nurse who discussed the study with you and gave you this leaflet. If you wish, you may also ask to speak to a member of the research team, listed below. Thank you for taking the time to read this information leaflet.

*Dr Janet Berrington*

*Dr Nicholas Embleton*

*Staff Nurse Julie Groombridge or local teams*

# Appendix 3: Consent form

**Chief Investigator:** Dr Janet Berrington

**Participant Number: Consent version: 1 dated 22/04/21**  **Please initial box**

1. I have read and understand the information sheet Version 1 dated 22/04/21 for the above study. I have had the opportunity to consider the information, ask questions and have had these answered satisfactorily. I also confirm I have a copy of the information sheet to keep.
2. I understand that my baby’s participation is voluntary and that I am free to withdraw my baby without giving any reason, and without my baby’s medical care or legal rights being affected.
3. I give permission to regulatory authorities or individuals from NHS Trusts and organisations to look at my baby’s health records in respect of the current study and any further research conducted in relation to it
4. I agree that my baby’s general practitioner may be informed of their participation in this study.

5. I understand that this consent form does not release the hospital or the research team from their liability.

6. I understand that my baby will be allocated at random to one of two breastmilk fortifiers given according to standard practices on the neonatal unit.

7. I agree that samples of stool and urine from my baby can be collected and analysed.

8. I agree to additional samples of blood (total volume <2.5mL) being taken for research purposes at the same time as other blood tests are needed.

9. I agree that the research team can keep in contact with me after discharge to see how my baby is progressing.

10. I agree that any stool, urine or blood samples that have already been collected but have not been analysed can be stored longer-term in the Great North Neonatal Biobank (HTA number 12534, ethics 21/NE/0024, IRAS project ID: 293413 )

Mother’s signature ……………………………………………………... Date...................................

Printed name ……………………………………...………………………

Witness signature ……………………………………………............. Date……………………............

Printed name of witness ……………………………………………..

Appendix 4

Suggested mineral and vitamin supplementation for CMBF and HMBF arms

Please note: adjustment should be made based on serum monitoring as routinely undertaken for preterm infants

| Arm | CMBF | HMBF | Age/point at commencement |
| --- | --- | --- | --- |
| Supplement |  |  |  |
| Dalivit | 0.3ml | 0.6ml | From full feeds |
| Vitamin K drops | Not needed | 50mcg | From full feeds |
| Iron | As per unit guidance | As per unit guidance | From 2 weeks after last transfusion or 4 weeks whichever later |
| Folate |  | 40mcg (0.5 ml) | From full feeds |

Appendix 5 Nutritional content of fortifiers

| **Nutrients** | **Unit** | **Mature human milk** 100 mL** | **NeoKare MMF/g** | Mature human milk 100 mL + 4 g (4 sachets) NeoKare MMF |
| --- | --- | --- | --- | --- |
| Energy | KJ Kcal | 289 69 | 17.99 4.30 | 360.96 86.20 |
| **Macronutrients** |  |  |  |  |
| Fat | g | 4.1 | 0.18 | 4.82 |
| Saturated fatty acids | g | ns | 0.08 | 0.33 |
| Carbohydrate | g | 7.2 | 0.4 | 8.80 |
| of which sugar (Lactose) | g | 7.2 | 0.34 | 8.56 |
| Protein | g | 1.3 | 0.27 | 2.38 |
| Salt (=sodiumx2.5) | g | 0.04 | 0.004 | 0.06 |
| **Vitamins** |  |  |  |  |
| Vitamin A | µg | 58 | 1.96 | 65.84 |
| Vitamin D | µg | trace | 0.018 | 0.072 |
| Vitamin E | mg | 0.34 | 0.0204 | 0.422 |
| Vitamin K | µg | ns | 0.024 | 0.096 |
| Vitamin C | mg | 4 | <0.001 | 4 |
| Thiamine (Vitamin B1) | mg | 0.02 | <0.001 | 0.02 |
| Riboflavin (Vitamin B2) | mg | 0.03 | 0.002 | 0.038 |
| Niacin (Vitamin B3) | mg | 0.2 | 0.044 | 0.376 |
| Vitamin B6 | mg | 0.01 | <0.0005 | 0.01 |
| Folic acid | µg | 5 | <0.1 | 5 |
| Vitamin B12 | µg | trace | 0.004 | 0.014 |
| Biotin (Vitamin B7) | µg | 0.7 | <100 | 0.7 |
| Pantothenic acid (Vit B5) | mg | 0.25 | NA | 0.25 |
| **Minerals** |  |  |  |  |
| Sodium | mg | 15 | 1.685 | 21.74 |
| Potassium | mg | 58 | 7.81 | 89.24 |
| Chloride | mg | 42 | 4.91 | 61.65 |
| Calcium | mg | 34 | 19 | 110.0 |
| Phosphorus | mg | 15 | 11 | 59.00 |
| Magnesium | mg | 3 | 1.57261 | 9.29 |
| Iron | mg | 0.07 | 0.003 | 0.08 |
| Zinc | mg | 0.3 | 0.036 | 0.44 |
| Copper | mg | 0.04 | 0.0035 | 0.054 |
| Manganese | mg | trace | 0.00022 | 0.001 |
| Fluoride | mg | ns | NA | ns |
| Selenium | µg | 1 | 0.203 | 1.812 |
| Chromium | µg | ns | <0.5 | <2 |
| Molybdenum | µg | ns | <0.10 | <0.4 |
| Iodine | µg | 7 | 0.73 | 9.94 |

**
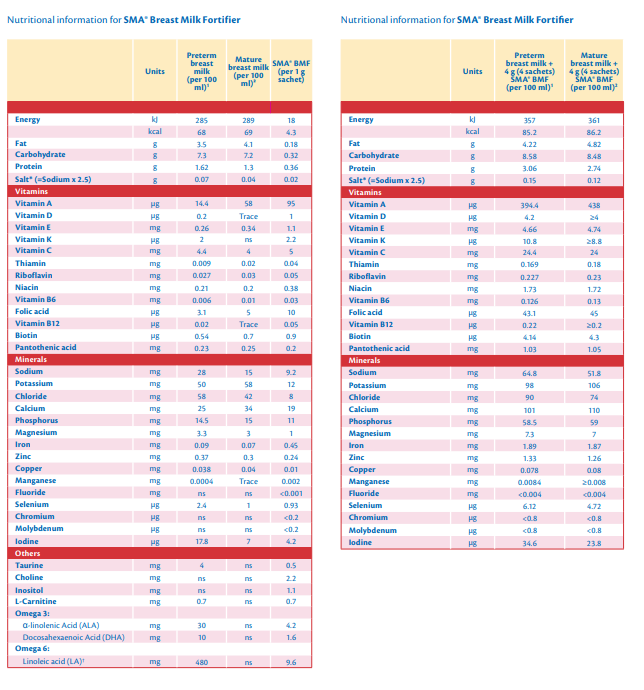
**

| **Please complete for infants with at least 5 days NBM, gut surgery or abdominal diagnosis resulting in death** | | | | | | |
| --- | --- | --- | --- | --- | --- | --- |
| **PUFFIN ID:** | | | | | | |
| Onset of the episode: day when abdominal concerns were raised and enteral feeds stopped (dd/mm/yy) | | | | | -- / -- / -- | |
| End of the episode: day when enteral feeds restarted (dd/mm/yy) or infant died if before feeds restarted | | | | | -- / -- / -- | |
| **What was the diagnosis? (please tick ONE box only)** | | | | | | |
| Suspected NEC not fulfilling criteria for stage II  (nil by mouth and antibiotics for minimum of  5 days)* | | |  | Focal intestinal perforation  (no NEC) | |  |
| Definite NEC: stage IIA or IIB* | | |  | Septic ileus | |  |
| Advanced NEC without perforation:  stage IIIA* | | |  | Dysmotility/meconium plug/milk plug | |  |
| Advanced NEC with perforation: stage IIIB* | | |  | Other (specify): | |  |
| **See overleaf for definitions of NEC** | | | | | | |
| **How was the diagnosis made? (please tick multiple boxes if appropriate)** | | | | | | |
| Clinically and radiologically | | |  | During surgery  Date: _ _ / _ _ / _ _ | |  |
| Histologically* | | |  | During post mortem  Date: _ _ / _ _ / _ _ | |  |
| ***If histology performed, the results:** | | | | | | |
| Confirm NEC |  | Do not confirm NEC |  | Confirm other diagnosis | |  |

Appendix 6 Reporting forms

**
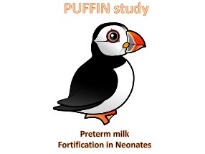
**PUFFIN Gut Form V1 150621

**
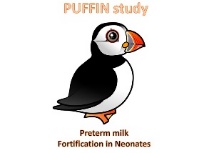
**PUFFIN LOS Form V1 150621

| Baby is diagnosed with NEC if at least one of the following clinical AND at least one of the following radiological features is present: | |
| --- | --- |
| Clinical (please tick) | Radiological (please tick) |
| Bilious gastric aspirates/emesis □  Abdominal distension □ Blood in the stool □ | Pneumatosis intestinalis □Hepatobiliary gas□  Pneumoperitoneum □ |

| **Bell’s stage** | **Systemic** | **Gastro-intestinal** | **Radiographic** |
| --- | --- | --- | --- |
| Stage IIA  (Definite NEC, mildly ill) | Increased desaturations Bradycardia  Temperature instability  Lethargy | Increased pre-feed gastric aspirate  Definite abdominal distension  Absent bowel sounds  Possible abdominal tenderness  Possibly bloody stools | Pneumatosis intestinalis |
| Stage IIB  (Definite NEC, moderately ill) | As IIA with  platelets <100x 10^9/^L  and/or metabolic acidosis:  BE< - 8mmol/L | Abdominal distension with definite tenderness  Possible abdominal wall oedema/erythema | As IIA with portal vein gas  Possible ascites |
| Stage IIIA  (Advanced NEC, bowel intact) | As IIB with mixed acidosis: pH<7.2  DIC: neutropaenia <1x10^9/^L  Severe apnoea  Hypotension requiring inotropes | Generalised peritonitis with severe tenderness and abdominal wall induration | As IIA with definite ascites |
| Stage IIIB  (Advanced NEC, bowel perforated) | As IIIA | As IIIA | As IIIA with pneumo -peritoneum |

| **Please complete for infants with at least 5 days antibiotics (or intent) unless being given for clear gut related pathology requiring gut form completion** |
| --- |
| **PUFFIN ID:** |

| Onset of the episode defined as start of the intravenous antibiotic/antifungal treatment for minimum of five days (dd/mm/yy): | | _ _ /_ _ /_ _ |
| --- | --- | --- |
| Was this episode microbiologically confirmed (in blood or CSF only)? | | YES □ NO □ |
| Name of organism: | Date of sample (dd/mm/yy): | Site:  Blood □ CSF □ |
| What IV antibiotic/antifungal was used for treatment of this episode? |  | |
| Duration of treatment (in days): |  | |

| **Definitions of confirmed and suspected LOS** |
| --- |
| **Microbiologically confirmed LOS** |
| 1. Onset of episode **more than 72 hours** after birth AND 2. Positive microbiological culture (blood or CSF) sampled with aseptic technique more than 72 hours after birth showing: potentially pathogenic bacteria (including coagulase negative Staphylococci, excluding skin contaminants and mixed flora) or fungi AND 3. Treatment (or intention to treat) for minimum of five consecutive days with intravenous antibiotics/antifungals after the above investigations performed.   *(If the infant doesn’t complete five days of treatment (for reasons such as transfer or death), but there exists documented intention to treat the episode for five days, this still meets the condition mentioned above)*  ***See over for clinically suspected LOS definition*** |
| **Clinically suspected LOS** |
| If the blood/CSF culture shows no growth/mixed flora or growth of skin contaminants AND infant is treated with intravenous antibiotics/antifungals for 5 consecutive days AND shows at least 3 or more of the following signs:   - Increase in oxygen requirement or ventilatory support - Increased frequency of apnea and bradycardia - Hypotension requiring treatment (volume, inotropes) - Poor peripheral perfusion (capillary refill time > 3s, mottled skin) - Irritability, lethargy or hypotonia - Ileus or enteral feeds intolerance (with or without abdominal distention) - Reduced urine output to < 1ml/kg/hour - Temperature instability - Glucose instability - Metabolic acidosis (base excess > -8 mmol/L and/or lactate > 2 mmol/L - C – reactive protein level > 15 mg/L - White cell count < 4 or >20 x 10^9^ cells/L or platelet count < 100 x 10^9^ cells/L |

| **Please specify the clinical/laboratory features leading to the decision to treat this episode:** | |
| --- | --- |
| Increase in oxygen requirement or ventilatory support |  |
| Increased frequency of apnea and bradycardia |  |
| Hypotension requiring treatment (volume, inotropes) |  |
| Poor peripheral perfusion (capillary refill time > 3s, mottled skin) |  |
| Irritability, lethargy or hypotonia |  |
| Ileus or enteral feeds intolerance (with or without abdominal distention) |  |
| Reduced urine output to < 1ml/kg/hour |  |
| Temperature instability |  |
| Glucose instability |  |
| Metabolic acidosis (base excess > -8 mmol/L and/or lactate > 2 mmol/L |  |
| C – reactive protein level > 15 mg/L |  |
| White cell count < 4 or > 20 x 10^9^ cells/L or platelet count < 100 x 10^9^ cells/L |  |
